# Distributed Fibre Optic Sensor-Based Continuous Strain Measurement along Semicircular Paths Using Strain Transformation Approach

**DOI:** 10.3390/s21030782

**Published:** 2021-01-25

**Authors:** Prashanth Nagulapally, Md Shamsuddoha, Ginu Rajan, Luke Djukic, Gangadhara B. Prusty

**Affiliations:** 1 ARC Training Centre for Automated Manufacture of Advanced Composites, School of Mechanical and Manufacturing Engineering, Sydney, NSW 2052, Australia; m.shamsuddoha@unsw.edu.au (M.S.); g.prusty@unsw.edu.au (G.B.P.); 2 School of Electrical, Computer, and Telecommunications Engineering, University of Wollongong, Wollongong, NSW 2522, Australia; 3 Omni Tanker Pty Ltd., 65-71 Hartley Road, Smeaton Grange, NSW 2567, Australia; luke.djukic@omnitanker.com

**Keywords:** distributed sensing, fibre optics, structural health monitoring, curved path strains

## Abstract

Distributed fibre optic sensors (DFOS) are popular for structural health monitoring applications in large engineering infrastructure because of their ability to provide spatial strain measurements continuously along their lengths. Curved paths, particularly semicircular paths, are quite common for optical fibre placement in large structures in addition to straight paths. Optical fibre sensors embedded in a curved path configuration typically measure a component of strain, which often cannot be validated using traditional approaches. Thus, for most applications, strain measured along curved paths is ignored as there is no proper validation tool to ensure the accuracy of the measured strains. To overcome this, an analytical strain transformation equation has been developed and is presented here. This equation transforms the horizontal and vertical strain components obtained along a curved semicircular path into a strain component, which acts tangentially as it travels along the curved fibre path. This approach is validated numerically and experimentally for a DFOS installed on a steel specimen with straight and curved paths. Under tensile and flexural loading scenarios, the horizontal and vertical strain components were obtained numerically using finite element analysis and experimentally using strain rosettes and then, substituted into the proposed strain transformation equation for deriving the transformed strain values. Subsequently, the derived strain values obtained from the proposed transformation equation were validated by comparing them with the experimentally measured DFOS strains in the curved region. Additionally, this study has also shown that a localised damage to the DFOS coating will not impact the functionality of the sensor at the remaining locations along its length. In summary, this paper presents a valid strain transformation equation, which can be used for transforming the numerical simulation results into the DFOS measurements along a semicircular path. This would allow for a larger scope of spatial strains measurements, which would otherwise be ignored in practice.

## 1. Introduction

Structural health monitoring (SHM) is the process of implementing a damage identification strategy for engineering structures and infrastructure [1]. SHM is a useful tool for ensuring the safety and integrity of a structure, detecting the evolution of damage, and estimating performance deterioration [2]. Early research work on SHM began in the 1970s; however, in the past decade, research on SHM accelerated due to the development of new sensors, electronic data storage, and computer data acquisition [3,4]. SHM consists of integrating the sensor devices with the structural components to extract continuous information related to the mechanical behaviour of a structure in various operational conditions [5,6]. Different types of sensors such as microelectromechanical systems (MEMS), accelerometers, optical fibres, vibration sensors, pressure-based sensors [7], and global positioning system (GPS) sensors [8] can be used during the SHM process for measuring a wide range of critical structural parameters, such as strain, temperature, displacement, pressure, and vibration [9]. Among all the structural parameters, strain can be considered as an important parameter, which can be utilised for SHM. Measuring strains on a structure has two advantages: firstly, strain directly reflects the deformation behaviour of a structure under loading, and secondly, strain is linked to the stress field, thereby any changes to a material stress field implies a change in the strain field [10,11]. Currently, surface strains on a structure are usually measured using foil strain gauges (FSG)/strain rosettes (SR), which are considered as a mature technology. However, FSGs are discrete sensors and can only provide localised strain values at locations close to the gauge [12], and moreover, they cannot be used for long-term SHM applications [13]. Alternatively, optical fibre sensors can be used to measure strains [14]. Optical fibre sensors, depending on the operating principle and construction, can be further divided into point, quasi-distributed, and distributed fibre optical sensors [15]. A discrete (point) optical fibre sensor such as a Fibre Bragg grating (FBG) sensor can provide strain measurement at a single location along the length of a fibre [16] and a significant amount of research work has been reported on SHM applications using FBG sensors [17]. Multiple FBG sensors can be multiplexed to form a quasi-distributed sensor. Contrary to point sensors and quasi-distributed sensors, distributed fibre optic sensors (DFOS) can provide one-dimensional spatial strain values continuously along their length with limited spatial resolution. Additionally, DFOSs are suitable for SHM during the operation of structures since they are capable of achieving the goals of diagnostics as well as condition monitoring [18]. Furthermore, they also exhibit many advantages including light weight, compact size, immune to electromagnetic interference, resistant to corrosion, and finally, the installation and operation of DFOS are simpler and more cost-effective compared to other sensors [19,20]. 

Due to superior sensing capabilities, DFOSs are gaining popularity among the research and industrial communities for sensing applications on various types of structures [21]. Shan et al. [10] employed DFOSs for monitoring strains on a complex curved composite structure. Distributed strain sensing along a high-performance composite hydrofoil with embedded DFOSs was performed by Maung et al. [12]. The sensing capabilities of a distributed fibre optic sensing system were evaluated by Davis et al. [22] by bonding DFOS on a centre fuselage of an ex-service aircraft under a full scale fatigue loading scenario. Three axis distributed strains were measured in 3D woven composite structures by Castellucci et al. [23]. Saidi and Gabor [24] embedded DFOSs into the core of textile reinforced cementitious matrix composites to assess the embedding capability, strength, and feasibility of strain measurements. Drake et al. [25] obtained the strain distributions from optical fibres arranged in three different configurations (spiral, grid, and rosette) on an aluminium cantilever beam subjected to tip load. The DFOS strain measurements in this study showed good agreement with strain gage measurements. Gifford et al. [26] demonstrated applicability of DFOS as a strain rosette by bonding a single DFOS in a circular loop configuration to a metal test sample. Meadows et al. [27] embedded DFOS in a double lap shear specimen and tensile tested them to obtain the strain response of the adhesive layer and to determine the impact of the sensor on the bond strength. Barker et al. [28] integrated a sensor system comprising fibre optic sensors and strain gauges on the rail and bridge members of Newmarket bridge in Canada to measure the strain experienced by the bridge during the passing of trains. Zhu et al. [29] proposed a smart carbon fibre reinforced polymer (CFRP) structure for distributed sensing with embedded DFOS. The CFRP package proposed in this paper provided mechanical protection to the optical fibre, enabled temperature–strain discrimination, and also facilitated the sensor’s installation to secure reliable measurements. The DFOS performance was investigated by Glisic et al. [30], who instrumented them on reinforced concrete elements. The DFOS characteristics investigated in this study include DFOS implementation methods, comparison and performance analysis of different bonding adhesives, and additionally, the fatigue performance of the DFOS sensors. 

**Figure 1:**
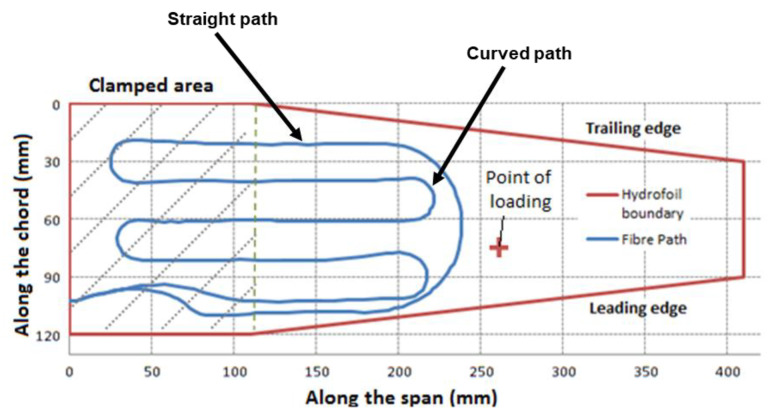
Layout of the distributed fibre optic sensor (DFOS) in straight path and semicircular path for measuring strains on a composite structure [12].

In all of these aforementioned research studies, the DFOSs were installed on the structures under testing and had two sections: straight paths and curved paths (turnaround region). The curved paths were primarily semicircular due to ease of placement, reproducibility, the fact that a semicircular loop of DFOS can also be used as a strain rosette [31], and moreover, a DFOS installed in a meandering pattern maximises the sensing region on a structure [32]. The schematic diagram of such fibre geometry is shown in Figure 1, which shows the DFOSs installed on the structures along with its two sections in two different research studies [3,4]. In all of these highlighted research studies, the DFOS strain measurements were validated using FSG measurements, finite element analysis (FEA), or a combination of both. Using FSGs for validating DFOS strain measurements is an expensive process in real structures as it requires multiple FSGs to be installed at various locations along the length of the fibre [33]. Furthermore, installing the FSGs is a complex and laborious process. FEA is considered as an inexpensive procedure for validating the DFOS strain measurements since it eliminates the need for complexities associated with the FSGs for installation. 

However, there are certain challenges associated with comparing FEA results with DFOS strain measurements. One of the primary challenges is a comparison of FEA results to the strain measurements along the curved path of the DFOS. In all the research studies conducted to date with the DFOS layout arrangement as shown in Figure 1, the DFOS strain measurements along the curved region have been ignored when validating with FEA results or they have been validated with the FSG results. Since the DFOS measures axial strains, comparing the FEA and DFOS strain measurements along a straight path is much simpler as it requires no additional strain transformation, whereas the axis of the DFOS is oriented tangentially along the curvature in a curved fibre path. Therefore, for comparison along a curved section of DFOS, the strain transformation of the FEA results is necessary. This strain transformation has to be undertaken at each location along the curved path of the DFOS using three strain components. To avoid the arduous task of strain transformation at each location along the curved region, the DFOS strain measurements have been validated by comparing them with the FEA strains along a straight path. This approach is effective as long as the DFOS is looped around noncritical areas of a structure. However, if a DFOS is looped around a critical stress concentration area of a structure such as a hole, bolt, or a rivet, it is imperative that the strain measurements are validated for accuracy around the critical stress concentration areas. According to the authors’ knowledge, no strain transformation equation has been presented in the literature, which can be used for simultaneous transformation of the normal and shear strain components at different locations along a semicircular curved path into a strain component measured by the DFOS along the curved path at the locations where normal and shear strain components have been obtained. 

**Figure 2:**
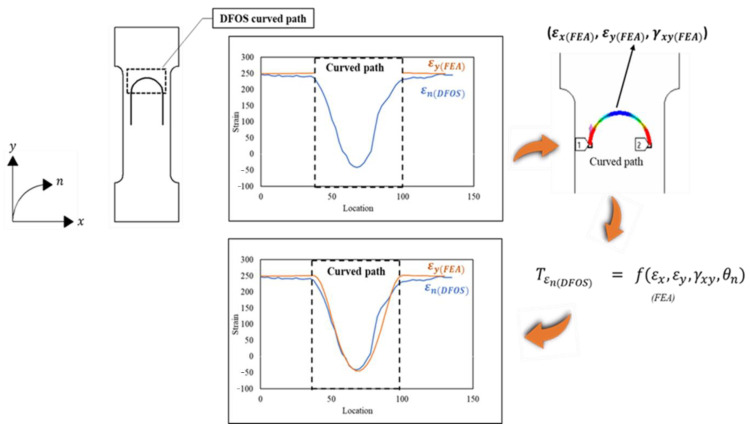
Illustration of the strain transformation method on a semicircular path of the distributed sensor.

In this paper, a strain transformation equation is presented for simultaneously transforming the horizontal, vertical, and shear strain components obtained at different locations along a semicircular path into a component of the strain measured by the DFOS along its curvature at those locations. The normal strain components $‘\varepsilon_{x}’$, $‘\varepsilon_{y}’$ and shear $‘\gamma_{xy}’$ strain components were obtained numerically using FEA and experimentally using five strain rosettes under two loading conditions at different locations along a semicircular path. Then, the numerically and experimentally obtained strain components were substituted into the proposed strain transformation equation for deriving the transformed strain value $\varepsilon_{n}$. Furthermore, the derived $\varepsilon_{n}$ values were validated by comparing them with the DFOS-measured $\varepsilon_{n}$ strain values. An illustration of the approach is depicted in Figure 2, which summarizes the procedure followed for transforming the horizontal and vertical strain components into a tangential strain component $\varepsilon_{n}$.

The study detailed in this paper was undertaken using numerical simulations and experimental work. The theoretical background and the development of the strain transformation equation are presented in Section 2, followed by the experimental program in Section 3, where descriptions of the FEA procedure, experimental program, sensors used and their installation, and load applied on the specimens are provided. In the results and discussion in Section 4, the strains obtained from the FEA, strain rosettes, and DFOS are presented along with a comparison between them and finally, the outcomes of this study are presented in the conclusions in Section 5. The strain transformation equation, which is the main contribution of this research to the paper, can be used for validating the DFOS strain measurements with the numerical simulation results along a semicircular section of the DFOS path. This will help to advance the field of distributed fibre optic sensing by increasing the accuracy and reliability of the strain measurements provided by the DFOS during SHM applications. 

## 2. Formulation of Transformation Equation

**Figure 3:**
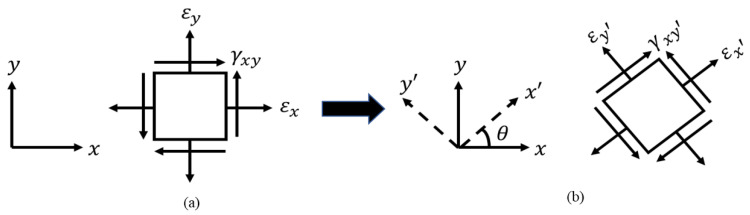
2D solid element in: (**a**) x−y coordinate system; (**b**) $x^{\prime }-y^{\prime }$ coordinate system.

The strain transformation equation presented in this paper is based on a 2D plane strain theory of elasticity. In a 3D strain state, a solid element has three normal and three shear strain components. In contrast, in a 2D plane strain condition, the strain normal to the x−y plane ($\varepsilon_{z}$), and the shear strain ($\gamma_{xz}$) and ($\gamma_{yz}$) are assumed to be zero. The non-zero normal strain components $\varepsilon_{x}$ and $\varepsilon_{y}$, and shear strain component $\gamma_{xy}$ on a 2D solid element in x−y coordinate system are shown in Figure 3. In the rotated 2D solid element shown in Figure 3, the axes $x^{\prime }$ and $y^{\prime }$ are oriented at certain angle θ with the x−y coordinate system. The strain transformation from the x−y coordinate system to $x^{\prime }-y^{\prime }$ can be achieved using the general 2D plane strain transformation in Equation (1).

(1) $$\left[\begin{array}{c} \varepsilon_{x^{\prime }}\\ \varepsilon_{y^{\prime }}\\ \gamma_{x^{\prime }y^{\prime }} \end{array}\right]=\left[\begin{array}{ccc} \cos^{2}\theta & sin^{2}\theta & sin\theta cos\theta \\ sin^{2}\theta & \cos^{2}\theta & -sin2\theta \\ -\sin\theta \cos\theta & \sin\theta \cos\theta & \cos2\theta \end{array}\right]\left[\begin{array}{c} \varepsilon_{x}\\ \varepsilon_{y}\\ \gamma_{xy} \end{array}\right]$$

**Figure 4:**
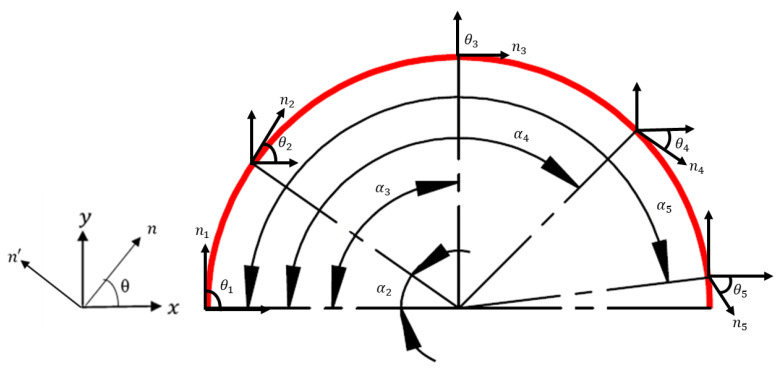
Schematic illustration of the semicircular geometry used in this study for strain transformation.

From past research studies [10,12,23,24,25,26,27,29,34], it can be seen that the most effective way of placing the DFOS sensor to increase the sensing region was to lay it in a straight line and in the form of a semicircular geometry. Therefore, the strain transformation equation that is presented here deals with a semicircular fibre path geometry. A schematic representation of the semicircular geometry, along whose curvature the strain transformation is undertaken, is shown in Figure 4. In the displayed x−y coordinate system, the x axis indicates the horizontal direction and the y axis denotes the vertical direction. The rotated axis which aligns tangentially with the curvature of the DFOS path is denoted by n. 

Equation (1) was simplified to provide only one strain component $\varepsilon_{n}$, which aligns with the n axis, as shown in Equation (2). The strain transformation can be undertaken at one location along the curvature of the DFOS using Equation (3). However, the equation was modified to perform the strain transformation simultaneously at five different locations along the curvature of the semicircular geometry. In Equation (4), $\varepsilon_{x1}-\varepsilon_{x5}$, $\varepsilon_{y1}-\varepsilon_{y5}$, and $\gamma_{xy1}-\gamma_{xy5}$ are the normal and shear strain components in the x−y plane at five locations along the curve. Angles $\theta_{1}-\theta_{5}$ are between the tangential axis *n* and horizontal axis *x*. Finally, the output $\varepsilon_{n1}$, $\varepsilon_{n2}$, $\varepsilon_{n3}$, $\varepsilon_{n4}$, and $\varepsilon_{n5}$ are the transformed values at five locations along the curvature of the semicircular geometry. Here, c and s are short forms of cosine and sine functions, respectively.

(2) $$\left[\begin{array}{c} \varepsilon_{n}\\ \varepsilon_{n^{\prime }}\\ \gamma_{nn^{\prime }} \end{array}\right]=\left[\begin{array}{ccc} cos^{2}\theta & sin^{2}\theta & sin\theta cos\theta \\ 0 & 0 & 0\\ 0 & 0 & 0 \end{array}\right]\left[\begin{array}{c} \varepsilon_{x}\\ \varepsilon_{y}\\ \gamma_{xy} \end{array}\right]$$

(3) $$\varepsilon_{n}=\varepsilon_{x}cos^{2}\theta \;+\varepsilon_{y}sin^{2}\theta +\gamma_{xy}\sin\theta cos\theta$$

(4) $$\left[\begin{array}{c} \varepsilon_{n1}\\ \varepsilon_{n2}\\ \varepsilon_{n3}\\ \varepsilon_{n4}\\ \varepsilon_{n5} \end{array}\right]=\left[\begin{array}{ccccccccccccccc} c^{2}\theta_{1} & s^{2}\theta_{1} & c\theta_{1}s\theta_{1} & 0 & 0 & 0 & 0 & 0 & 0 & 0 & 0 & 0 & 0 & 0 & 0\\ 0 & 0 & 0 & c^{2}\theta_{2} & s^{2}\theta_{2} & c\theta_{2}s\theta_{2} & 0 & 0 & 0 & 0 & 0 & 0 & 0 & 0 & 0\\ 0 & 0 & 0 & 0 & 0 & 0 & c^{2}\theta_{3} & s^{2}\theta_{3} & c\theta_{3}s\theta_{3} & 0 & 0 & 0 & 0 & 0 & 0\\ 0 & 0 & 0 & 0 & 0 & 0 & 0 & 0 & 0 & c^{2}\theta_{4} & s^{2}\theta_{4} & c\theta_{4}s\theta_{4} & 0 & 0 & 0\\ 0 & 0 & 0 & 0 & 0 & 0 & 0 & 0 & 0 & 0 & 0 & 0 & c^{2}\theta_{5} & s^{2}\theta_{5} & c\theta_{5}s\theta_{5} \end{array}\right][\begin{array}{c} \varepsilon_{x1}\\ \varepsilon_{y1}\\ \gamma_{xy1}\\ \varepsilon_{x2}\\ \varepsilon_{y2}\\ \gamma_{xy2}\\ \varepsilon_{x3}\\ \varepsilon_{y3}\\ \gamma_{xy3}\\ \varepsilon_{x4}\\ \varepsilon_{y4}\\ \gamma_{xy4}\\ \varepsilon_{x5}\\ \varepsilon_{y5}\\ \gamma_{xy5} \end{array}]$$

## 3. Experimental Program

**Figure 5:**
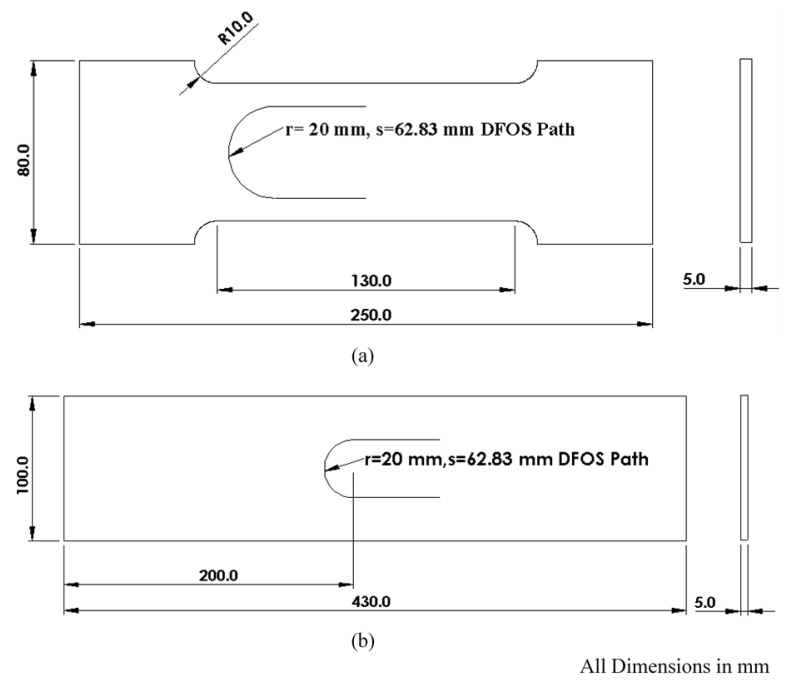
Geometrical dimensions of the specimens for: (**a**) Tensile test; (**b**) Flexural test.

For deriving the $\varepsilon_{n}$ strain measurements using Equation (4), the normal and shear strains were measured numerically and experimentally on a 5 mm thick flat steel specimen under two different loading scenarios: (i) Tensile and (ii) Flexural. In each loading scenario, three incremental loads were applied on the specimen in each run. The dimensions of the specimen used for tensile loading are shown in Figure 5a, and the dimensions of the specimen used for flexural testing are shown in Figure 5b. Initially, numerical analysis was performed using commercial FEA software, ANSYS workbench 19.1, and a later experiment was performed under two loading conditions for validation.

### 3.1. Finite Element Analysis (FEA)

The FEA was performed on the 3D model of a flat steel specimen under tensile and flexural loads. A linear elastic material model was used. The material properties of the structural steel used in this analysis were Young’s modulus (*E*) of 205 GPa, Poisson’s ratio (*µ*) of 0.3, and a Yield strength (*σ_y_*) of 250 MPa. In FEA, meshing of the geometry determines the quality and accuracy of the strain results. For computational efficiency and accuracy, a finer mesh with an element size of 3 mm was used in the critical sections of the 3D FE models for the two loading cases. The tensile loaded specimen was meshed with a combination of 30,910 quad and tetrahedron elements. The gauge length, which was the critical area of the specimen, was meshed with quad elements, and remaining surfaces mainly gripping the section were meshed with tetrahedron elements. In contrast, the geometry of the flexural loading specimen was flat without any shoulders, therefore it was meshed with 62,086 quad elements. A semicircular path with radius of 20 mm was created on the specimen to replicate the curved semicircular region of the DFOS. In the tensile loading scenario, three loads of 5, 10, and 15 kN were applied on one end of the specimen and a fixed support boundary condition constraining all the degrees of freedom was specified on the other end of the specimen. In contrast, for the flexural tests, the specimen was under the cantilever loading condition with a fixed support boundary condition applied on one end of the specimen and loading was applied at the other end of the specimen. Three varying flexural loads of 50, 75, and 100 N were applied on the specimen during the flexural testing. For both load cases, normal and shear strain components were extracted at ten locations along the curve at angles $\alpha_{1}-\alpha_{10}$, which are shown in Table 1. The angles $\alpha_{1}-\alpha_{10}$ are measured from the horizontal axis ‘x’ as shown in Figure 4. As the axis ‘n’, which is shown in Figure 4, travels along the length of the curve, it makes an angle ‘$\theta_{n}$’ with the horizontal axis. The angles $\theta_{1}-\theta_{10}$ between the axis ‘n’ and the ‘x’ axis at the ten measured locations are displayed in Table 2. The normal and shear strain components obtained from FEA along with the angles $\theta_{1}-\theta_{10}$ were substituted into Equation (4) for deriving the $\varepsilon_{n}$ values. 

### 3.2. Specimens for Experiments

The flat steel specimens used in the experiments had similar dimensions to FEA geometries. Under tensile load, FEA had predicted almost identical strains on both the surfaces of the specimen. Hence, for validating the FEA strain measurements, five triaxial 0°/45°/90° stacked strain rosettes were used along with the DFOS during the tensile test. A single rosette provided the $\varepsilon_{x}$, $\varepsilon_{y}$, and $\gamma_{xy}$ components of strain at one specific location. These components (15 from 5 rosettes) of the strain were substituted into Equation (4) for deriving the $\varepsilon_{n}$ values. The DFOS, which was bonded on the other side of the specimen, directly provided the $\varepsilon_{n}$ values along its length. However, during the flexural testing, the strains on both the surfaces of the specimen are dissimilar, so only DFOS was used. 

#### 3.2.1. Strain Rosette (SR) Installation 

**Figure 6:**
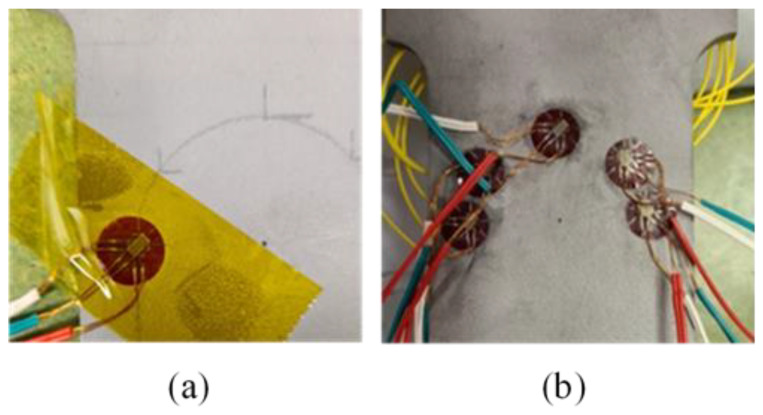
Details of strain rosettes: (**a**) triaxial strain rosette being placed; (**b**) strain rosettes installed on the semicircular path.

Five rosettes supplied by Tokyo Measuring Instruments Lab were used in this study. The rosettes were installed at five locations to form a semicircle with a radius of 20 mm. The locations where the rosettes were installed on the curve correspond to locations $\alpha_{1}$, $\alpha_{3}$, $\alpha_{5}$, $\alpha_{7}$, and $\alpha_{9}$, which are detailed in Table 1. The gauge length and resistance were 3 mm and 120 Ω, respectively. Before installing the rosettes, the surface of the specimen was abraded and cleaned with isopropanol to remove all sharp edges and particulates, which could influence the strain measurements. Finally, the rosettes were bonded with cyanoacrylate adhesive at the marked locations. The surface of the specimen with rosettes installed is shown in Figure 6. 

#### 3.2.2. Instrumentation of the Distributed Fibre Optic Sensor (DFOS)

**Figure 7:**
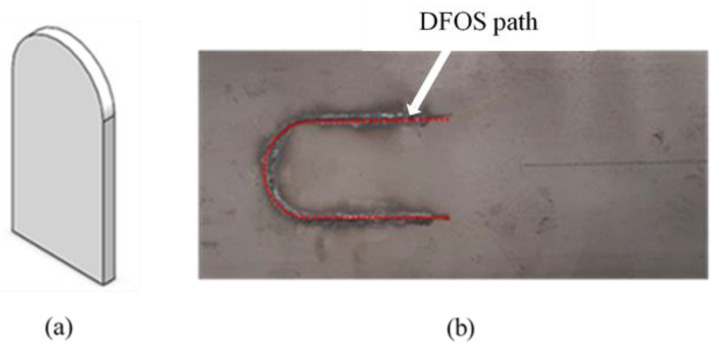
Preparation of specimen: (**a**) Guide used for mounting the DFOS; (**b**) DFOS path on the surface of the specimen.

On the tensile test specimen, numerical simulations had shown equal strain levels on the two surfaces of the specimen. For this reason, the DFOS was installed on the opposite surface of the specimen to where strain rosettes were installed. In contrast, for the flexural test specimen, numerical simulations had shown that one side of the specimen was under tension, while the other surface of the specimen was under compression. To be consistent with the tensile testing, the DFOS was installed on the surface, which experienced tension under applied load. A single mode polyimide-coated optical fibre with a diameter of 150 µm was used in this study. Before bonding the DFOS, the surfaces of the steel specimens were cleaned with isopropanol. Using a semicircular guide with radius of 20 mm placed on the surface of the specimen, markings were made for installing the DFOS. Finally, the DFOS was bonded along the markings with cyanoacrylate adhesive, as shown in Figure 7. A Luna innovations ODiSI-B^®^ distributed fibre optic sensor interrogator was used for measuring the strains along the length of the DFOS. The interrogator had a gauge length of 5 mm with a sampling spacing of 2.6 mm. 

#### 3.2.3. Experiments for Tensile and Flexural Loading

**Figure 8:**
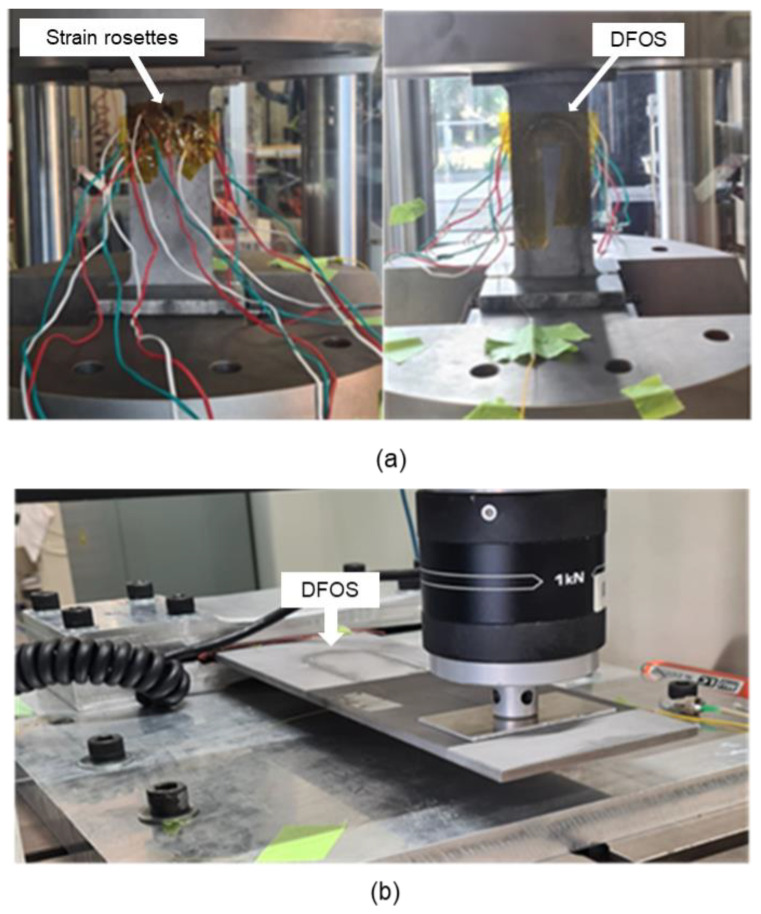
Specimens along with its sensors during testing: (**a**) Tensile loading; (**b**) Flexural loading.

The tensile and flexural loads were applied on the steel specimens using an Instron universal testing machine with 50 and 1 kN load cells, respectively. Initially, a tensile test was carried out, followed by flexural testing. In the two loading scenarios, the load applied on the specimens was similar to what was applied during the FEA. The specimen, along with its sensors fixed in the jaws of the tensile testing machine, is shown in Figure 8a. Under flexural loading, a jig was used for fixing one end of the specimen and load was applied on the other end of the specimen, as shown in Figure 8b. 

## 4. Results and Discussion

### 4.1. Strains Predicted from FEA

**Figure 9:**
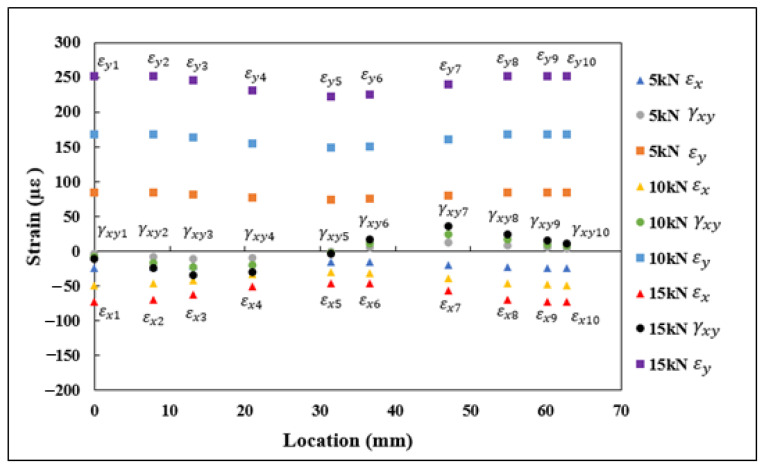
$\varepsilon_{x1}–\varepsilon_{x10}$, $\varepsilon_{y1}-\varepsilon_{y10}$, and $\gamma_{xy1}–\gamma_{xy10}$ normal and shear strains obtained through FEA under tensile loading.

**Figure 10:**
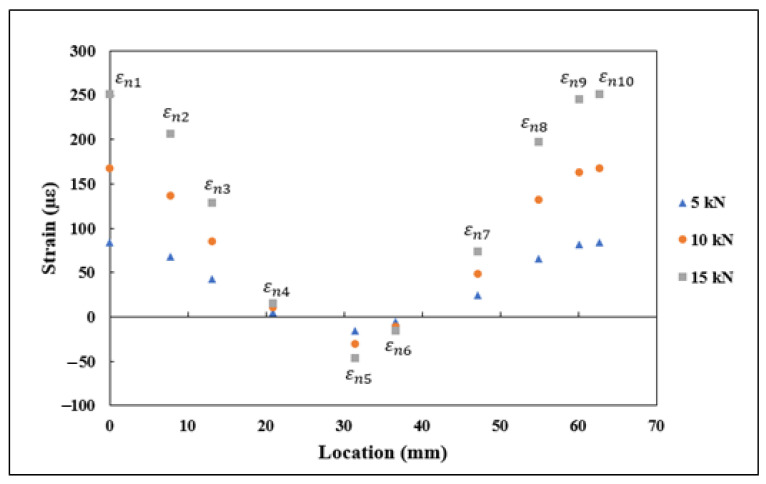
$\varepsilon_{n1}-\varepsilon_{n10}$ transformed strains under tensile loading.

The normal and shear strain components under tensile and flexural loads, $\varepsilon_{x}$, $\varepsilon_{y}$, and $\gamma_{xy}$, were extracted at ten different locations along the length of the curve, outlined in Table 1. The normal and shear strains for 5, 10, and 15 kN tensile loads using FEA are shown in Figure 9. In Figure 9, it can be observed that the strains oriented with the vertical axis were under tension, while the strains oriented with the horizontal axis were under compression, and the shear strain components were transitioning from compression to tension along the curvature of the semicircle. The normal and shear strains ($\varepsilon_{x1}-\varepsilon_{x10}$, $\varepsilon_{y1}-\varepsilon_{y10}$, $\gamma_{x1}-\gamma_{x10}$) and angles at different locations ($\theta_{1}-\theta_{10})$ are substituted into Equation (4) for evaluating the transformed strains ($\varepsilon_{n1}-\varepsilon_{n10})$. The transformed strains $\varepsilon_{n1}-\varepsilon_{n10}$ under the tensile load are shown in Figure 10. The results from the plot indicate that the $\varepsilon_{n}$ values were under tension at the start of the curve, then transitioned into compressive strains at the midpoint of the curve, and finally, at the end of the curvature, the strains were under tension.

**Figure 11:**
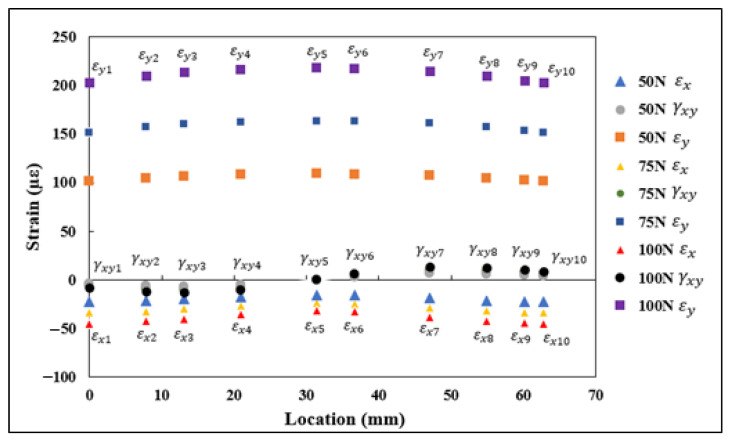
$\varepsilon_{x1}–\varepsilon_{x10}$, $\varepsilon_{y1}–\varepsilon_{y10}$, and $\gamma_{xy1}–\gamma_{xy10}$ normal and shear strains obtained through FEA under flexural loading.

**Figure 12:**
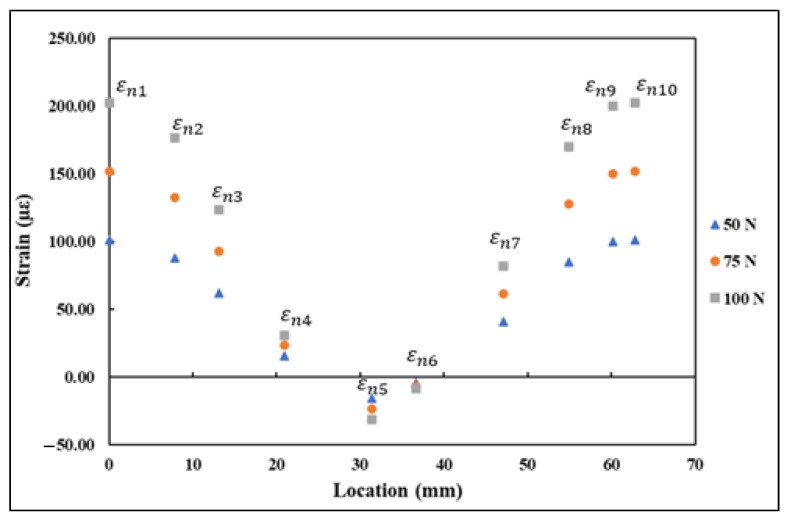
$\varepsilon_{n1}–\varepsilon_{n10}$ transformed strains under flexural loading.

On the specimen loaded under flexure, the DFOS was on the tensile surface. To be consistent with the experimental measurements, in FEA, the $\varepsilon_{x}$, $\varepsilon_{y}$, and $\gamma_{xy}$ values were extracted on a similar surface which had undergone tension due to applied load. The normal and shear strains $\varepsilon_{x1}-\varepsilon_{x10}$, $\varepsilon_{y1}-\varepsilon_{y10}$, and $\gamma_{xy1}-\gamma_{xy10}$ from FEA under 50, 75, and 100 N flexural load are shown in Figure 11. The strains shown in Figure 11 for flexural loading followed a similar trend to the strains obtained under tensile loading. The vertical strain components $\varepsilon_{y}$ were under tension, while the horizontal strain components $\varepsilon_{x}$ were under compression, and the shear strain components $\gamma_{xy}$ transitioned from compression to tension from the start to the end of the curve. The normal and shear strain components and the $\theta_{1}-\theta_{10}$, shown in Table 2, were substituted into the Equation (4) for finding the transformed strain values $\varepsilon_{n}$. The derived strain values $\varepsilon_{n1}-\varepsilon_{n10}$ using the proposed strain transformation equation are shown in Figure 12. The strains shown in Figure 12 also followed a similar trend to the $\varepsilon_{n}$ values derived under tensile loading, which are shown in Figure 10, transitioning from tension to compression and to tension from start to the end of the curve.

### 4.2. Strains Measured Using SR

**Figure 13:**
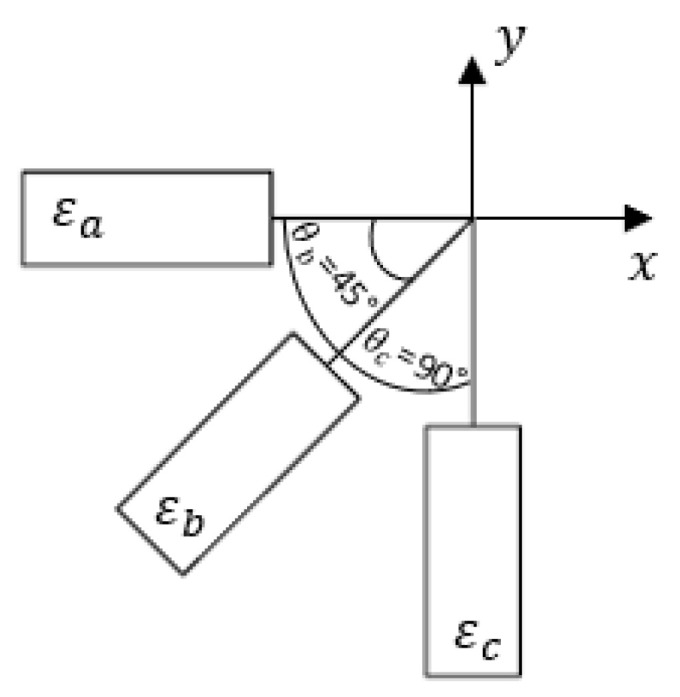
Illustration of the triaxial strain rosette used in this study.

**Figure 14:**
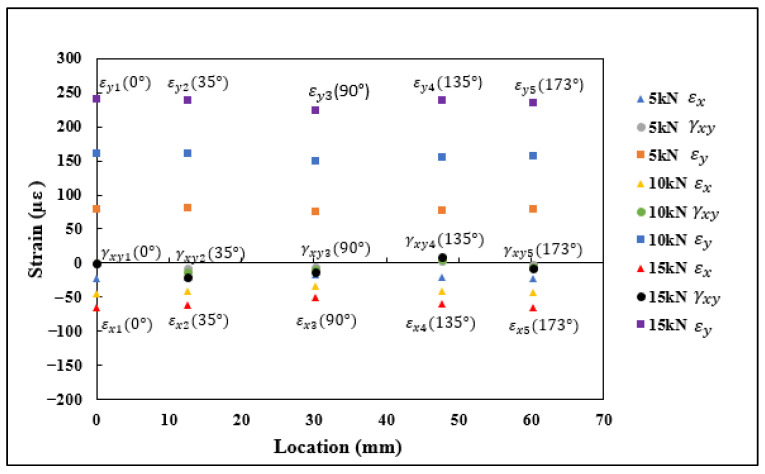
$\varepsilon_{x1}-\varepsilon_{x5}$, $\varepsilon_{y1}–\varepsilon_{y5}$, and $\gamma_{xy1}–\gamma_{xy5}$ strain measurements obtained using strain rosettes.

**Figure 15:**
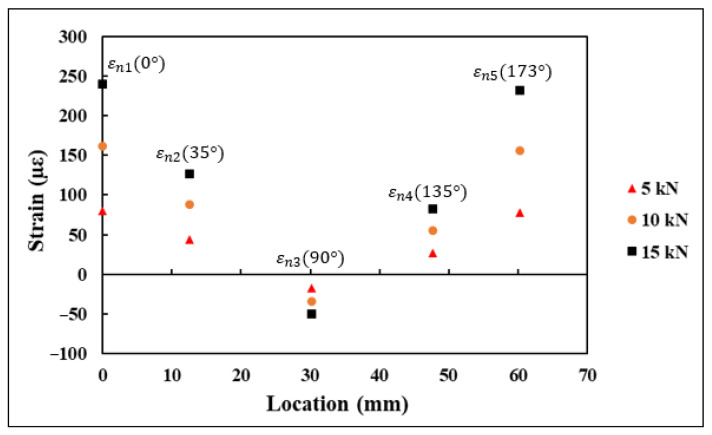
$\varepsilon_{n1}–\varepsilon_{n5}$ transformed strains derived from strain rosette measurements.

Each 0°/45°/90° stacked SR provided three strain measurements, namely $\varepsilon_{a}$, $\varepsilon_{b}$, and $\varepsilon_{c}$, aligned along the axis, as shown in Figure 13. These strain measurements were substituted into rosette transformation (Equation (5)) for obtaining the normal and shear strain components $\varepsilon_{x}$, $\varepsilon_{y}$, and $\gamma_{xy}$. The strain measurements $\varepsilon_{x1}-\varepsilon_{x5}$, $\varepsilon_{y1}-\varepsilon_{y5}$, and $\gamma_{xy1}-\gamma_{xy5}$ obtained from the five rosettes under the application of 5, 10, and 15 kN tensile load are shown in Figure 14. A similar procedure to the one used with the FEA results was used with the strains measured using rosettes for $\varepsilon_{n1}-\varepsilon_{n5}$. The transformed strain values $\varepsilon_{n1}–\varepsilon_{n5}$ derived from the rosette measurements are presented in Figure 15. The strain measurements, which are shown in Figure 14 and Figure 15, had a close correlation both qualitatively and quantitively to the strains shown in Figure 9 and Figure 10, which are obtained numerically under the tensile loading scenario.

(5) $$\left[\begin{array}{c} \varepsilon_{a}\\ \varepsilon_{b}\\ \varepsilon_{c} \end{array}\right]=\left[\begin{array}{ccc} cos^{2}\left(\theta_{a}\right) & sin^{2}\left(\theta_{a}\right) & cos\theta_{a}\;sin\theta_{a}\\ cos^{2}\left(\theta_{b}\right) & sin^{2}\left(\theta_{b}\right) & cos\theta_{b}\;sin\theta_{b}\\ cos^{2}\left(\theta_{b}\right) & sin^{2}\left(\theta_{c}\right) & cos\theta_{c}\;sin\theta_{c} \end{array}\right]\left[\begin{array}{c} \varepsilon_{x}\\ \varepsilon_{y}\\ \gamma_{xy} \end{array}\right]$$

### 4.3. Strains Monitored Using DFOS

**Figure 16:**
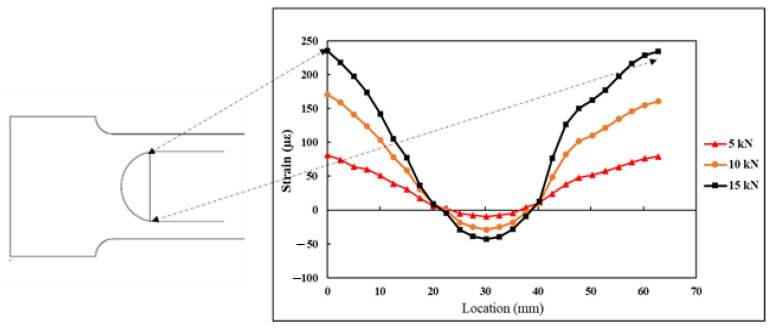
Strains measured by the DFOS under tensile load.

The DFOS was adhesively bonded on the specimen surface in a semicircular form, with a radius of curvature r=20 mm and an arc length of 62.83 mm. The DFOS measured the strains at 2.6 mm intervals along its length. The strain values $\varepsilon_{n}$, measured by the DFOS along its length under 5, 10, and 15 kN tensile load, are shown in Figure 16. The start and end of the curve are also highlighted in Figure 16. From the plot, it can be seen that at the start of the curve, the strains on the specimen are maximum and tensile, and at the centre of the curve, the specimen strains are at maximum compression. 

**Figure 17:**
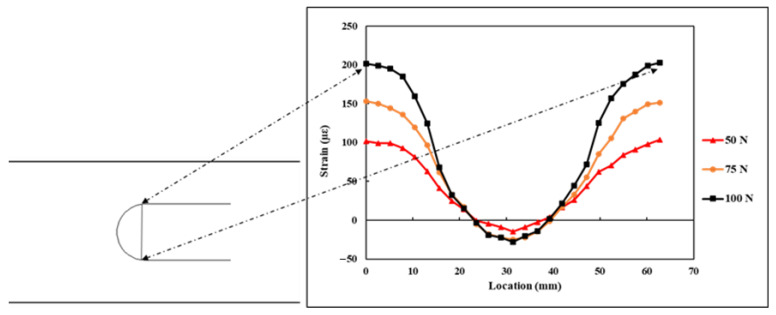
Strains measured by the DFOS under flexural load.

The DFOS strain measurements ($\varepsilon_{n}$) on the flat steel specimen under the application of three varying flexural loads are shown in Figure 17. The strains measured by the DFOS along the curvature during the application of flexural load had a similar trend to that of strains measured during the tensile loading. The strains are tensile and maximum at the start and end of the curve, and at the centre of the curve, the specimen strains are maximum compression strain. Gifford et al. [26], in their research paper, have reported that for a uniaxial load, the strain distribution along a circular DFOS loop is in the form of a sinusoidal pattern. The semicircular DFOS strain measurements, which are obtained in this study for both tensile and flexural load, followed a similar pattern to what was highlighted in the research study by Gifford et al. [26], however with a phase difference. The strains measured by the DFOS for both the load cases were within the elastic limit of the steel which correlated with the applied load.

### 4.4. Comparison of the Strain Measurements between FEA, DFOS, and SR

**Figure 18:**
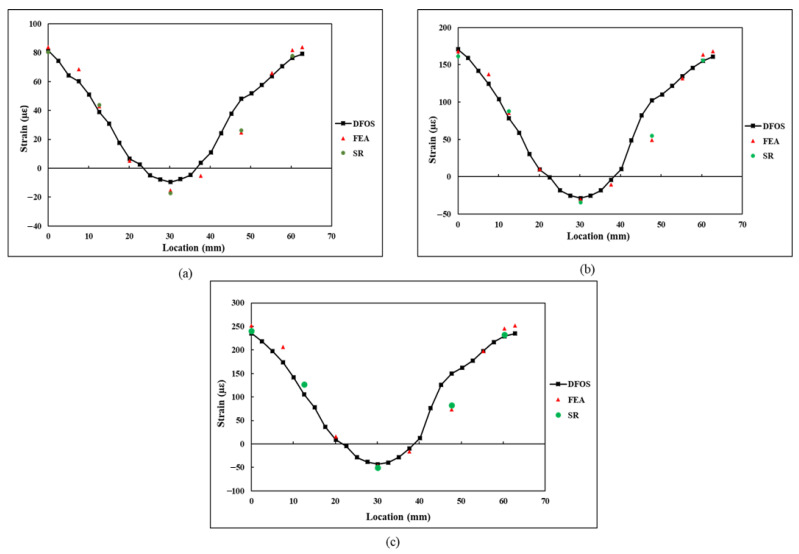
Comparison of the $\varepsilon_{n1}–\varepsilon_{n10}$ values obtained in tensile specimen at a loading of: (**a**) 5 kN; (**b**) 10 kN; (**c**) 15 kN.

The $\varepsilon_{n}$ strain values derived by the FEA and rosettes (denoted by SR) were compared with the DFOS strain measurements for validation. A comparison of the strain measurements $\varepsilon_{n}$ under tensile load is presented in Figure 18. In the plot shown in Figure 18, it can be observed from the plot that the strain increased linearly with an increase in applied load. Furthermore, the strains were within the elastic strain limit of the steel. The $\varepsilon_{n}$ values derived from the FEA and strain rosettes are in close agreement with each other. The FEA and strain rosettes results are also in close agreement with the DFOS results. However, at the location $\alpha_{7}$, at an arc length of 47.1 mm, significant deviation can be observed in the $\varepsilon_{n}$ values measured by the DFOS. 

**Figure 19:**
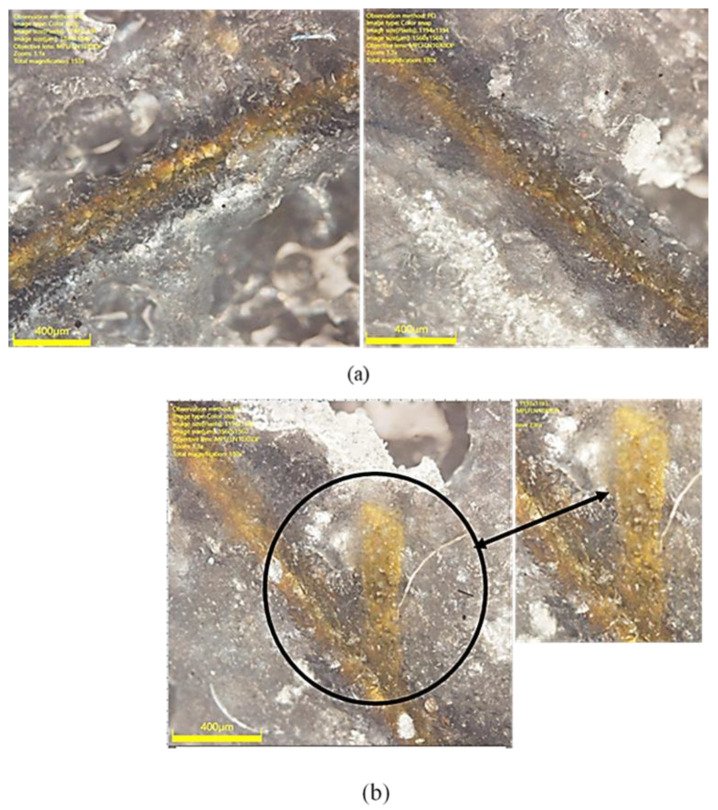
Microscopic images of the surface bonded DFOS: (**a**) intact DFOS coating; (**b**) damaged DFOS coating.

Initially, it was theorized that the large difference in strain values could be due to the localised imperfect bonding of the DFOS on the surface of the specimen. To identify the flaws in the sensor bonding, the DFOS was examined under optical microscopy. A commercial Digital Olympus DSX510 microscope with 13.5X optical zoom and 30X with digital zoom was used for microscopic examination of the DFOS coating. Microscopic examination showed that while there was no flaw in the DFOS bonding, the DFOS polyimide coating was damaged at the location where the sensor measured high strain reading. The intact DFOS coating at two different locations on the fibre is shown in Figure 19a, and the damaged DFOS coating is displayed in Figure 19b. The damage to the DFOS coating may have occurred during the sensor installation process and may have contributed to the high localised strain readings. A comparison of the strain difference between the coated and uncoated fibre for the same applied stress was carried out by Okabe et al. [35], through calculations and experiments. The results from this study showed that an uncoated fibre measures high strains than a coated fibre, which provided a smoothened strain distribution. The influence of different DFOS coatings on the strain measurements has been further highlighted in the previous research studies conducted by Weisbrich and Holschemacher [36] and Oliveira et al. [37]. At the remaining locations, the $\varepsilon_{n}$ values obtained through FEA, strain rosettes, and DFOS correlated with each other. The strong correlation of the DFOS readings, strain rosettes readings, and FEA results at locations of approximately 10 mm away from the damaged location indicates that the DFOS can continue to function with integrity with a point of localised damage.

**Figure 20:**
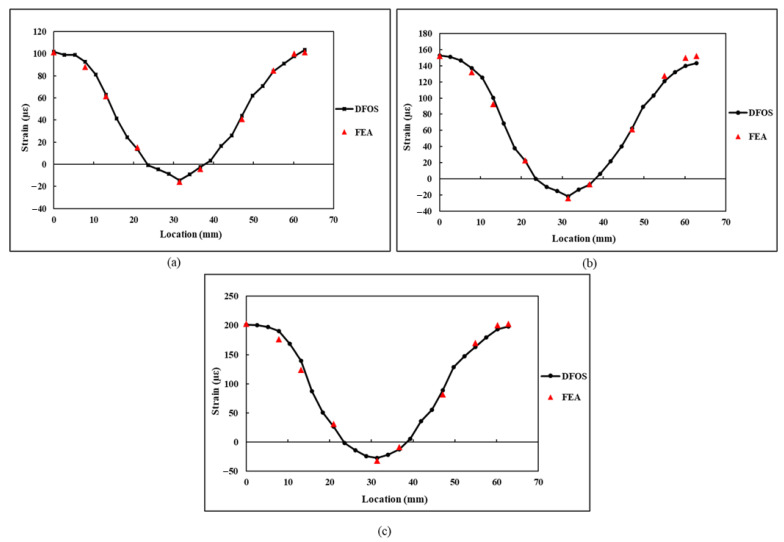
Comparison of the $\varepsilon_{n1}–\varepsilon_{n10}$ values obtained in flexural specimen at a loading of: (**a**) 50 N; (**b**) 75 N; (**c**) 100 N.

A comparison of the derived strain values using the strain transformation equation and measured strain values using the DFOS under flexural loading is presented in Figure 20. Since no strain rosettes were used during the flexural testing, the derived $\varepsilon_{n}$ values were only compared with the $\varepsilon_{n}$ measurements of the DFOS. From the results, a close correlation between the derived strain values and measured strain values can be observed. Furthermore, the difference between the strain values for flexural loading is less than that of the tensile load. During the flexural testing, good adhesion and an intact sensor resulted in the reduction in deviation between the DFOS and FEA strain values. 

The comparison results shown in Figure 18 and Figure 20 under tensile and flexural loading scenarios confirm that the strain transformation equation which is proposed in this paper can be used for transforming the normal and shear strain components along a semicircular path into a component of strain measured by the DFOS along that semicircular path. The equation is derived based on characteristic parameters for a semicircle, which can be translated to any scalable structures as long as the DFOS path is semicircular and the minimum diameter specified by the manufacturer is maintained. Furthermore, on the assumption that behaviour of the structure is elastic and the adhesion of the sensor to the substrate is intact, any loading condition either static or dynamic in nature would not affect the strain measurements because the dynamic loading is a time-dependent loading and DFOS would provide accurate response under such loading conforming the transformation. Although this study was performed on a smaller specimen under static loading, the proposed strain transformation equation can be valid for larger structures subjected to dynamic loading when the DFOS is bonded in a semicircular path around the critical areas of a structure. Furthermore, based on the semicircular strain field results obtained in this research study and also the strain field along a full circular loop from a previous investigation conducted by Gifford et al. [26], it can be asserted that the DFOS strain field follows a distinct sinusoidal pattern along a circular curve, which is independent of the structure size and loading. However, the strain transformation equation presented in this paper is valid only when the DFOS is bonded in a semicircular path with a radius of curvature as specified by the manufacturer of the DFOS sensor. Any deviations along the curvature of the semicircle may lead to inaccuracies in the strain transformation. Furthermore, the DFOS must be free from defects and also, the surface of the structure under test must be flat and free from any curvature.

## 5. Conclusions

Distributed fibre optic sensors (DFOS) are popular for structural health monitoring applications in large engineering structures and infrastructure because of their ability to provide spatial strain measurements continuously along their length. The DFOS is laid on the surface of a structure for measuring strains in the form of straight lines connected between semicircular loops. Usually, the strains along the curved region of the DFOS are neglected when validating the DFOS strain measurements with the computational simulations. In this study, an analytical strain transformation equation was presented to transform the horizontal and vertical strain components obtained along a semicircular path into a strain component which acts tangentially as it travels along that semicircular path. The main conclusions of this study can be summarised as follows:

- The transformed strain values derived through the strain transformation equation correlated well with the experiments under tensile and flexural loading scenarios.
- The DFOS strain field along a circular or semicircular curve follows a distinct sinusoidal pattern which is independent from the structure size and loading.
- A localised damage to the DFOS coating can influence the strain measurements at that location. However, deviations in DFOS readings are localised to the point of damage event under certain conditions, which is further evidence of the suitability of the DFOS in SHM applications.
- Overall, this study has successfully demonstrated that the proposed strain transformation equation can be used for transforming the horizontal and vertical strain components obtained through FEA along a semicircular geometry into a tangential component of the strain measured by the distributed fibre optic sensor along the curved semicircular paths.

## Figures and Tables

**Table 1:** Locations for transformation along the arc length of the curve.

Location	α (°)	Arc Length (mm)
$\alpha_{1}$	0	0
$\alpha_{2}$	20	6.98
$\alpha_{3}$	35	12.21
$\alpha_{4}$	58	20.24
$\alpha_{5}$	90	31.41
$\alpha_{6}$	108	37.69
$\alpha_{7}$	135	47.12
$\alpha_{8}$	158	55.15
$\alpha_{9}$	173	60.38
$\alpha_{10}$	180	62.83

**Table 2:** $\theta_{1}–\theta_{10}$ between the n-axis and *x*-axis.

Location	θ (°)
$\theta_{1}$	90
$\theta_{2}$	70
$\theta_{3}$	55
$\theta_{4}$	32
$\theta_{5}$	0
$\theta_{6}$	−18
$\theta_{7}$	−45
$\theta_{8}$	−68
$\theta_{9}$	−83
$\theta_{10}$	−90

## Data Availability

The raw data required to reproduce these findings cannot be shared at this time due to time limitations but will be made available through the UNSW data repository.

## References

[1] Farrar C.R., Worden K. (2007). An introduction to structural health monitoring. Philos. Trans. R. Soc. A Math. Phys. Eng. Sci..

[2] Ou J., Li H. (2009). Structural health monitoring research in China: Trends and applications. Structural Health Monitoring of Civil Infrastructure Systems.

[3] Cawley P. (2018). Structural health monitoring: Closing the gap between research and industrial deployment. Struct. Health Monit..

[4] Brownjohn J.M.W. (2007). Structural health monitoring of civil infrastructure. Philos. Trans. R. Soc. A Math. Phys. Eng. Sci..

[5] Giraldo C.D.M. (2018). Development of Optical Fiber Sensors for the Structural Health Monitoring in Aeronautical Composite Structures. Ph.D. Thesis.

[6] Boller C. State-of-the-art in structural health monitoring for aeronautics. Proceedings of the International Symposium on NDT in Aerospace.

[7] Tibaduiza Burgos D.A., Gomez Vargas R.C., Pedraza C., Agis D., Pozo F. (2020). Damage Identification in Structural Health Monitoring: A Brief Review from Its Implementation to the Use of Data-Driven Applications. Sensors.

[8] Guzman-Acevedo G.M., Vazquez-Becerra G.E., Millan-Almaraz J.R., Rodriguez-Lozoya H.E., Reyes-Salazar A., Gaxiola-Camacho J.R., Martinez-Felix C.A. (2019). GPS, Accelerometer, and Smartphone Fused Smart Sensor for SHM on Real-Scale Bridges. Adv. Civ. Eng..

[9] Moreno-Gomez A., Perez-Ramirez C.A., Dominguez-Gonzalez A., Valtierra-Rodriguez M., Chavez-Alegria O., Amezquita-Sanchez J.P. (2018). Sensors Used in Structural Health Monitoring. Arch. Comput. Methods Eng..

[10] Shan Y., Xu H., Zhou Z., Yuan Z., Xu X., Wu Z. (2019). State sensing of composite structures with complex curved surface based on distributed optical fiber sensor. J. Intell. Mater. Syst. Struct..

[11] Glisic B., Yao Y., Tung S.T.E., Wagner S., Sturm J.C., Verma N. (2016). Strain Sensing Sheets for Structural Health Monitoring Based on Large-Area Electronics and Integrated Circuits. Proc. IEEE.

[12] Maung P.T., Prusty B.G., Rajan G., Li E., Phillips A.W., St John N.A. (2017). Distributed strain measurement using fibre optics in a high performance composite hydrofoil. ICCM Int. Conf. Compos. Mater..

[13] Yehia S., Abudayyeh O., Abdel-Qader I., Zalt A., Meganathan V. (2008). Evaluation of sensor performance for concrete applications. Trans. Res. Rec..

[14] Rajan G., Prusty G. (2016). Structural Health Monitoring of Composite Structures Using Fibre Optic Methods.

[15] Wu T., Liu G., Fu S., Xing F. (2020). Recent progress of fiber-optic sensors for the structural health monitoring of civil infrastructure. Sensors.

[16] Di Sante R. (2015). Fibre optic sensors for structural health monitoring of aircraft composite structures: Recent advances and applications. Sensors.

[17] Subash Chandra Mukhopadhyay (2011). New Developments in Sensing Technology for Structural Health Monitoring.

[18] Ramakrishnan M., Rajan G., Semenova Y., Farrell G. (2016). Overview of fiber optic sensor technologies for strain/temperature sensing applications in composite materials. Sensors.

[19] Kaur A., Jothibasu S., Anandan S., Du Y., Dhaliwal G., Huang J., Chandrashekhara K., Watkins S. (2018). Strain monitoring using distributed fiber optic sensors embedded in carbon fiber composites. Sensors and Smart Structures Technologies for Civil, Mechanical, and Aerospace Systems.

[20] Glisic B., Inaudi D. (2011). Development of method for in-service crack detection based on distributed fiber optic sensors. Struct. Health Monit..

[21] Levin K. (2008). Durability of Embedded Fibre Optic Sensors in Composites. Ph.D. Thesis.

[22] Davis C., Knowles M., Rajic N., Swanton G. (2016). Evaluation of a Distributed Fibre Optic Strain Sensing System for Full-Scale Fatigue Testing. Procedia Struct. Integr..

[23] Castellucci M., Klute S., Lally E.M., Froggatt M.E., Lowry D. (2013). Three-axis distributed fiber optic strain measurement in 3D woven composite structures. Ind. Commer. Appl. Smart Struct. Technol..

[24] Saidi M., Gabor A. (2019). Use of distributed optical fibre as a strain sensor in textile reinforced cementitious matrix composites. Meas. J. Int. Meas. Confed..

[25] Drake D.A., Sullivan R.W., Wilson J.C. (2018). Distributed strain sensing from different optical fiber configurations. Inventions.

[26] Gifford D.K., Froggatt M.E., Sang A.K., Kreger S.T. (2012). Multiple fiber loop strain rosettes in a single fiber using high resolution distributed sensing. IEEE Sens. J..

[27] Meadows L., Sullivan R.W., Vehorn K. Distributed optical sensing in composite laminate adhesive bonds. Proceedings of the 57th AIAA/ASCE/AHS/ASC Structures, Structural Dynamics, and Materials Conference.

[28] Barker C., Hoult N.A., Le H., Tolikonda V. Evaluation of a railway bridge using distributed and discrete strain sensors. Proceedings of the International Conference on Smart Infrastructure and Construction 2019 (ICSIC): Driving Data-Informed Decision-Making.

[29] Zhu P., Xie X., Sun X., Soto M.A. (2019). Distributed modular temperature-strain sensor based on optical fiber embedded in laminated composites. Compos. Part B Eng..

[30] Glisic B., Huston D.R., Navarra G., Barrias A., Casas J.R., Villalba S. (2019). SHM of Reinforced Concrete Elements by Rayleigh Backscattering DOFS. Front. Built Environ..

[31] Gifford D.K., Sang A.K., Kreger S.T., Froggatt M.E. (2010). Strain measurements of a fiber loop rosette using high spatial resolution Rayleigh scatter distributed sensing. Fourth Eur. Work Opt. Fibre Sens..

[32] Nathan I., Meyendorf N., Nathan I., Meyendorf N. (2019). Handbook of Advanced Nondestructive Evaluation.

[33] Silva A.L., Varanis M., Mereles A.G., Oliveira C., Balthazar J.M. (2019). A study of strain and deformation measurement using the Arduino microcontroller and strain gauges devices. Rev. Bras. Ensino Fis..

[34] Bao Y., Chen G. (2015). Strain distribution and crack detection in thin unbonded concrete pavement overlays with fully distributed fiber optic sensors. Opt. Eng..

[35] Okabe Y., Tanaka N., Takeda N. (2002). Effect of fiber coating on crack detection in carbon fiber reinforced plastic composites using fiber Bragg grating sensors. Smart Mater. Struct..

[36] Weisbrich M., Holschemacher K. (2018). Comparison between different fiber coatings and adhesives on steel surfaces for distributed optical strain measurements based on Rayleigh backscattering. J. Sens. Sens. Syst..

[37] De Oliveira R., Schukar M., Krebber K., Michaud V. Distributed Strain Measurement in Cfrp Structures By Embedded Optical Fibres: Influence of the Coating. Proceedings of the 6th International Conference on Composite Structures, ICCS 16.

